# Development of Magnetic Particle–Based Chemiluminescence Immunoassays for Detecting PCV2 VLP‐ and Rep‐Specific Antibodies and Their Combined Use for Immune Evaluation and Replication‐Associated Exposure Assessment

**DOI:** 10.1155/tbed/3733817

**Published:** 2026-05-31

**Authors:** Mingxi Gou, Xiaoqing Song, Jie Fan, Zhixiong Chen, Zihao Kuang, Mengyao Lu, Zhongxin Fan, Meng Ge

**Affiliations:** ^1^ College of Veterinary Medicine, Hunan Agricultural University, Changsha, China, hunau.edu.cn; ^2^ Hunan Engineering Technology Research Center of Veterinary Drugs, Hunan Agricultural University, Changsha, 410128, Hunan, China, hunau.edu.cn; ^3^ Hunan Animal Disease Prevention and Control Center, Changsha, 410128, Hunan, China

**Keywords:** chemiluminescence immunoassay (CLIA), porcine circovirus 2 (PCV2), Rep protein, replication-associated exposure, serological surveillance, virus-like particles (VLPs)

## Abstract

Porcine circovirus 2 (PCV2) remains a major swine pathogen, and interpretation of capsid protein (Cap)–based serology is challenging in vaccinated populations because vaccine‐induced Cap antibodies overlap with replication‐associated serological signals. To address this issue, we developed two fully automated magnetic particle–based chemiluminescence immunoassays (CLIAs), including a Cap virus‐like particle (VLP)–based assay (VLPs‐CLIA) for evaluating Cap antibody response intensity and a replication‐associated protein (Rep)–based assay (rRep‐CLIA) for assessing replication‐associated serological exposure or pressure. We produced self‐assembled PCV2 Cap VLPs and recombinant Rep in a prokaryotic system and covalently coupled them to carboxylated magnetic beads for fully automated CLIAs. VLPs‐CLIA was calibrated and reported in activity units (U), whereas rRep‐CLIA was interpreted as a threshold‐based qualitative assay using a relative light unit (RLU) cut‐off. Analytical performance and agreement with reference enzyme‐linked immunosorbent assays (ELISAs) were evaluated using longitudinal and field serum samples. VLPs‐CLIA detected antibodies up to 1:25,600, approximately twofold higher than the reference ELISA (1:12,800), with 95.68% agreement (Kappa = 0.876, *p*  < 0.001). rRep‐CLIA agreed well with Rep‐ELISA (94.86%; Kappa = 0.846, *p*  < 0.001) and provided a complementary signal consistent with replication‐associated exposure. Both assays showed good precision (coefficients of variation [CVs] ranging from 2.83% to 6.94%) and good reagent stability. In field samples, VLPs‐CLIA tracked postvaccination Cap kinetics, whereas Rep seropositivity increased with age and exposure background, suggesting heterogeneous replication–associated pressure among herds. The two assays together support complementary herd‐level interpretation of vaccine‐induced Cap immunity and replication‐associated serological exposure, particularly in Cap‐vaccinated populations.

## 1. Introduction

Porcine circovirus 2 (PCV2) is a nonenveloped, circular single‐stranded DNA virus and is among the smallest known animal viruses [1]. As the primary etiological agent of porcine circovirus–associated diseases (PCVAD), PCV2 is associated with clinical syndromes such as postweaning multisystemic wasting syndrome (PMWS) and porcine dermatitis and nephropathy syndrome (PDNS), resulting in substantial economic losses to the global swine industry [2–6]. Historically, PCV2a and PCV2b were predominant, whereas PCV2d has become the major circulating genotype in recent years [7, 8]. Therefore, a PCV2d‐derived capsid protein (Cap) sequence was selected in this study to improve antigenic representativeness with respect to the currently prevalent field strains. In parallel, replication‐associated protein (Rep) was included because it is a relatively conserved replication‐associated nonstructural antigen among PCV2 strains and may provide complementary serological information beyond Cap‐based vaccination responses.

The Cap of PCV2 is the major immunogenic protein and is capable of self‐assembling in vitro into virus‐like particles (VLPs) [9]. These VLPs preserve native conformational epitopes while lacking infectivity, making them suitable antigens for serological assays and subunit vaccines [9, 10]. Accordingly, Cap‐ or VLP‐based enzyme‐linked immunosorbent assays (ELISAs) have been widely used to detect PCV2‐specific antibodies [11].

However, because most commercial PCV2 subunit vaccines are Cap‐based, vaccine‐induced Cap antibodies overlap with infection‐associated signals, making serology difficult to interpret in vaccinated populations [12]. This overlap can bias herd‐level infection assessment, confound vaccine efficacy evaluation, and hinder eradication‐oriented surveillance.

In contrast to Cap, the nonstructural Rep is essential for PCV2 genome replication and transcriptional regulation and is expressed early during infection [13, 14]. Previous studies indicate that Rep is relatively conserved across porcine circoviruses (PCV1 and PCV2) and that Rep‐specific antibodies are associated with active viral replication during natural infection, whereas Cap‐based vaccination does not appear to induce detectable Rep antibody responses [15–17]. Collectively, these features support Rep serology as a complementary marker that can help interpret infection‐associated serological signals in Cap‐vaccinated populations when considered alongside Cap profiles and herd epidemiological contexts [18]. Accordingly, Cap was selected as the antigen for evaluating serological immune responses against the major immunogenic structural protein, whereas Rep was included as a relatively conserved replication‐associated antigen to provide complementary information beyond Cap‐based vaccination responses.

From a diagnostic perspective, conventional ELISAs, although well‐established, are limited by relatively lower sensitivity, labor‐intensive procedures, and restricted automation. In contrast, fully automated magnetic particle–based chemiluminescence immunoassays (CLIAs) offer high sensitivity, a wide dynamic range, streamlined workflows, and compatibility with high‐throughput testing. Accordingly, CLIA has become an important platform in modern immunodiagnostics [19] and shows considerable potential for veterinary infectious disease surveillance and diagnosis [20–23].

Therefore, this study aimed to develop two complementary automated CLIAs to support interpretation of PCV2 serology in vaccinated populations: a VLP‐based assay to evaluate vaccine‐associated Cap antibody responses and a Rep‐based assay (rRep‐CLIA) to provide complementary information on replication‐associated serological exposure or pressure. To this end, we developed two complementary CLIAs: a Cap VLP–based assay (VLPs‐CLIA) to quantify vaccine‐induced Cap antibody response intensity and a rRep‐CLIA to indicate replication‐associated serological pressure. Through systematic comparison, methodological validation, and evaluation of combined use, we established a dual‐marker framework to assess serological immune status (Cap/VLPs) and replication‐associated serological pressure (Rep), providing support for epidemiological investigations, control decisions, and eradication‐oriented surveillance.

## 2. Materials and Methods

### 2.1. Serum Samples

All serum samples are summarized in Table 1 and include reference sera, an assay establishment/validation set (*n* = 200), and a field application set (*n* = 1534). Reference sera comprised PCV2 standard positive/negative sera and sera positive for other common swine pathogens for specificity testing. The field set included longitudinal, cross‐sectional, and slaughterhouse sera. The assay establishment/validation set (*n* = 200) was used for assay optimization and receiver operating characteristic (ROC)–based cut‐off determination, as detailed in Section 2.9.

**Table 1:** Information on clinical serum samples.

Source/region	Farm ID	Category and immune status	Sampling design	Number of samples
Experimental pigs	A	Vaccinated pigs (longitudinal monitoring)	From 1 week before vaccination to 10 weeks after vaccination; 8 sampling time points	32
	F	Nonvaccinated pigs (longitudinal monitoring)	2–24 weeks of age; 12 sampling time points	82
Changsha, Hunan	G	Nonvaccinated pigs (longitudinal monitoring)	4–30 weeks of age; 16 sampling time points	112
	B	Clinical sera (longitudinal monitoring)	1–18 weeks of age; 10 sampling time points	100
	1	Sera from boars, sows, and gilts	Single sampling	Boars (8), sows (8), gilts (8); total 24
	2	Sera from boars, sows, and gilts	Single sampling	Boars (20), sows (57), gilts (21); total 98
Zhuzhou, Hunan	D	Clinical sera (longitudinal monitoring)	2–24 weeks of age; 12 sampling time points	180
	C	Vaccinated pigs (vaccinated at 5 weeks of age; longitudinal monitoring)	5–21 weeks of age; 7 sampling time points	195
	3	Sera from boars and sows	Single sampling	Boars (3), sows (25); total 28
	4	Sera from boars and sows	Single sampling	Boars (13), sows (47); total 50
Loudi, Hunan	E	Clinical sera (longitudinal monitoring)	4–24 weeks of age; 8 sampling time points	31
Loudi, Hunan	5	Sera from sows and gilts	Single sampling	Sows (13), gilts (7); total 20
	6	Sera from boars, sows, and gilts	Single sampling	Boars (1), sows (9), gilts (5); total 15
Xiangtan, Hunan	7	Breeding pig sera	Single sampling	25
Yongzhou, Hunan	8	Sow sera	Single sampling	25
	9	Sera from boars, sows, and gilts	Single sampling	Boars (7), sows (23), gilts (13); total 43
Changde, Hunan	10	Sow sera	Single sampling	80
	11	Breeding pig sera	Single sampling	50
Huaihua, Hunan	12	Sera from boars, sows, and gilts	Single sampling	Boars (20), sows (52), gilts (23); total 95
Chenzhou, Hunan	13	Breeding pig sera	Single sampling	106
Shaoyang, Hunan	14	Sow sera	Single sampling	10
Hunan Slaughterhouse 1	15	Finishing pig sera	Single sampling	65
Hunan Slaughterhouse 2	16	Finishing pig sera	Single sampling	42
Hunan Slaughterhouse 3	17	Finishing pig sera	Single sampling	26
Total				**1534**

*Note:* Farms A–G were included in longitudinal monitoring, while farms 1–17 were included in cross‐sectional monitoring. Immune status refers to vaccination background at the time of sampling. The value in bold indicates the total number of samples.

### 2.2. Reagents and Instruments

Carboxylated magnetic beads (1 μm in diameter) were purchased from Nanjing Dongna Biotechnology Co., Ltd. Acridinium ester (AE) was obtained from Helisen Biotechnology Co., Ltd. Goat antiporcine IgG antibody was purchased from Solarbio Science & Technology Co., Ltd. The concentrated washing buffer, pretrigger solution, and trigger solution required for chemiluminescence detection were provided by Fapon Biotech Inc. All CLIA measurements were performed on a Shine i1000 fully automated CLIA analyzer (Fapon Biotech Inc.).

### 2.3. Expression and Purification of PCV2 rRep and Cap Protein

Based on the PCV2 ORF1 (GenBank accession number AY188355) and ORF2 (GenBank accession number KY947581.1) sequences, the corresponding coding regions were synthesized and cloned into pET‐28a(+) to generate pET28a‐rRep and pET28a‐Cap2dΔ41, respectively. The pET28a‐Cap2dΔ41 construct was an N‐terminally truncated PCV2d Cap expression construct rather than the full‐length ORF2 construct, in which amino acids 1–41 were deleted. The deleted segment corresponds to the arginine‐rich nuclear localization signal (NLS)–containing region. This truncation was used to improve soluble expression in *Escherichia coli* and to facilitate VLP assembly while preserving the major antigenic region of Cap.

The recombinant plasmids were transformed into *E. coli* BL21 (DE3)–competent cells. Expression conditions were optimized by varying the induction temperature and IPTG concentration. For Cap2dΔ41, soluble expression was evaluated at 16, 25, and 37°C with 1.0 mM IPTG, and the protein used for purification was expressed at 37°C with 1.0 mM IPTG. For rRep, soluble expression was evaluated at 16, 25, and 37°C with either 0.5 or 1.0 mM IPTG, and the protein used for purification was expressed at 16°C with 1.0 mM IPTG.

After induction, bacterial cells were harvested and disrupted by ultrasonication. The lysates were centrifuged to separate soluble and insoluble fractions, and the supernatants were collected for purification. The recombinant proteins were purified using Ni^2+^‐affinity chromatography. Briefly, the clarified supernatants were loaded onto the Ni^2+^ column, and unbound proteins were collected as the flow‐through fraction. The column was then washed and eluted stepwise with buffers containing increasing concentrations of imidazole. For Cap2dΔ41, fractions eluted with 50, 100, 250, and 500 mM imidazole were collected. For rRep, fractions eluted with 50, 100, 250, and 500 mM imidazole were also collected. All collected fractions, including the bacterial supernatant, pellet, flow‐through, and imidazole elution fractions, were analyzed by SDS‐PAGE. The identity of the purified target proteins was further confirmed by Western blotting.

### 2.4. Assembly and Characterization of PCV2 Cap VLPs

Purified Cap2dΔ41 protein was dialyzed against assembly buffer (100 mM NaH_2_PO_4_, 500 mM NaCl, 100 mM KCl, 10 mM Tris‐HCl, 5% glycerol, 0.5% Triton X‐100, and 0.1 mM PMSF; pH 7.4) at 4°C for 4 h to promote self‐assembly into VLPs. VLP morphology was examined by transmission electron microscopy (TEM), and VLP concentration was determined using a bicinchoninic acid (BCA) assay.

### 2.5. Preparation of Magnetic Bead–Antigen Conjugates

Magnetic bead–antigen conjugates were prepared according to the manufacturer’s instructions for the carboxylated magnetic bead coupling kit. Briefly, 2 mg of carboxylated magnetic beads was activated using EDC/NHS chemistry and coupled with rRep protein or VLPs. After coupling, the beads were blocked with a bovine serum albumin (BSA)–containing buffer and subsequently resuspended in storage buffer consisting of 50 mM phosphate buffer (PB), 0.9% NaCl, 2 mg/mL BSA, 0.05% Tween 20, 0.1% PC300, 0.1% BND 10, and 0.1% PEG 6000 (pH 7.4). The final bead concentration was adjusted to 5 mg/mL, and conjugates were stored at 4°C until use. Before use, the working suspension was diluted to 0.3 mg/mL.

### 2.6. Labeling of Detection Antibody

Goat antiporcine IgG was labeled with an AE using a commercial labeling kit. The antibody and AE were reacted at an optimized molar ratio of 15:1 at room temperature under light‐protected conditions. After completion of the labeling reaction, the conjugates were purified by dialysis. The labeled antibody was resuspended in buffer containing 50% glycerol and stored at −20°C in the dark. Before use, the labeled antibody was diluted to the optimal working concentration using storage buffer consisting of 50 mM MES, 0.9% NaCl, 2 mg/mL BSA, 0.05% Tween 20, 0.1% PC 300, 0.1% BND 10, and 0.1% PEG 6000 (pH 6.5).

### 2.7. Establishment of the CLIA

The CLIAs were established on the Shine i1000 fully automated CLIA analyzer. Briefly, serum samples were prediluted in PBS and then automatically diluted by the analyzer. The diluted samples were sequentially incubated with antigen‐coupled magnetic beads and AE‐labeled secondary antibodies, followed by washing steps. Finally, chemiluminescent substrates were added, and signals were recorded as relative light units (RLUs).

Key parameters were optimized, including (i) antigen‐to‐bead coupling ratios (5, 10, and 20 μg/mg), (ii) dilution of AE‐labeled antibody (1:125–1:1000), (iii) serum dilution (1:50–1:800), and (iv) reaction format and incubation time (one‐step, delayed one‐step, or two‐step; 5–20 min per step).

### 2.8. Standard Curves and Quality Control

A laboratory‐preserved high‐titer PCV2–positive serum was serially diluted (1:100–1:102,400) and calibrated using commercial ELISA kits. Samples yielding results close to the cut‐off were defined as borderline sera and assigned 40 activity units (U). Based on this calibration, six dilution points (1:102,400, 1:25,600, 1:6400, 1:1600, 1:400, and 1:100) were selected to construct the standard curve, corresponding to 0, 10, 40, 160, 640, and 2560 U, respectively. In addition, one calibrated PCV2 Cap–negative serum and one Cap–positive serum (PCV2 Cap Cal1 and Cal2) and one Rep–negative serum and one Rep–positive serum (PCV2 Rep Neg1 and Pos2) were included as quality control samples in each run. A quantitative standard curve was established for VLPs‐CLIA, whereas rRep‐CLIA was interpreted primarily according to the RLU cut‐off determined by ROC analysis. Accordingly, VLPs‐CLIA was used as a calibrated assay with U‐based output, whereas rRep‐CLIA was interpreted primarily as a qualitative assay based on the ROC‐derived RLU cut‐off; raw RLU values were used only for signal visualization and not as calibrated quantitative measurements.

### 2.9. Methodological Performance Evaluation

The analytical performance was evaluated as follows.

Cut‐off determination: For ROC analysis, a validation panel of 200 sera was used, comprising 119 reference‐positive and 81 reference‐negative samples for the Cap assay and 33 reference‐positive and 167 reference‐negative samples for the Rep assay, as classified by the previously reported reference ELISA methods. ROC curves were generated in MedCalc, and the cut‐off values were determined by maximizing the Youden index. These sera were used for cut‐off determination and were partially overlapped with the samples used during assay optimization.

Analytical sensitivity: High‐titer positive sera were serially diluted and tested using the established CLIAs in parallel with previously reported Cap‐ELISA [24] and Rep‐ELISA [25].

Specificity: Sera positive for PCV2 and other common swine pathogens, including classical swine fever virus (CSFV), porcine reproductive and respiratory syndrome virus (PRRSV), porcine epidemic diarrhea virus (PEDV), *Mycoplasma hyopneumoniae* (Mhp), *Actinobacillus pleuropneumoniae* (APP), and porcine circovirus 3 (PCV3), were tested to evaluate potential cross‐reactivity.

Repeatability: Intra‐assay (*n* = 10) and interassay (*n* = 4) precision were assessed, and coefficients of variation (CVs) were calculated. Precision was evaluated descriptively by calculating intra‐assay and interassay CVs. Because no single universal CV threshold applies across all veterinary serological assays, precision was interpreted in a fit‐for‐purpose manner. In addition, the observed precision values were considered in the context of commonly used bioanalytical and ligand‐binding assay validation frameworks [26, 27].

Stability: Accelerated stability was assessed by incubating quality control samples at 37°C and testing them on days 0, 3, and 7.

Agreement analysis: A total of 370 clinical serum samples were tested in parallel using the established CLIAs and the previously reported Cap‐ELISA and Rep‐ELISA.

### 2.10. Evaluation of Clinical Application

To evaluate the field applicability, two complementary strategies were employed.

Longitudinal dynamic monitoring: Herds selected for longitudinal dynamic monitoring included farms with well‐defined vaccination backgrounds, namely, vaccinated farms A and C and nonvaccinated farms F and G, together with clinically monitored herds with unknown vaccination backgrounds, namely, farms B, D, and E. Serum samples collected at multiple time points were tested in parallel using VLPs‐CLIA and rRep‐CLIA, together with the previously reported Cap‐ELISA and Rep‐ELISA methods. Antibody dynamics obtained by different methods within the same herds were compared to assess the ability of CLIAs to monitor antibody kinetics, detect serological changes potentially associated with replication‐related exposure, and maintain consistency with ELISA trends.

Cross‐sectional screening and epidemiological analysis: To assess performance in large‐scale screening and to preliminarily characterize regional epidemiological status, serum samples were collected from core breeding farms and slaughterhouses across multiple regions of Hunan Province, comprising 669 breeding herd sera and 133 slaughterhouse sera. All samples were tested using a combined VLPs‐CLIA and rRep‐CLIA approach.

This design had two objectives: (i) to evaluate vaccine coverage and overall serological (humoral) immune status in core breeding herds based on Cap antibody positivity measured by VLPs‐CLIA and (ii) to assess replication‐associated exposure among herds based on Rep antibody positivity measured by rRep‐CLIA, which reflects replication‐associated exposure rather than vaccination. Ultimately, combined VLPs‐CLIA and rRep‐CLIA results were used to support herd‐level sero‐epidemiological assessment and epidemiological surveillance.

## 3. Results

### 3.1. Preparation and Characterization of Antigens

**Figure 1:**
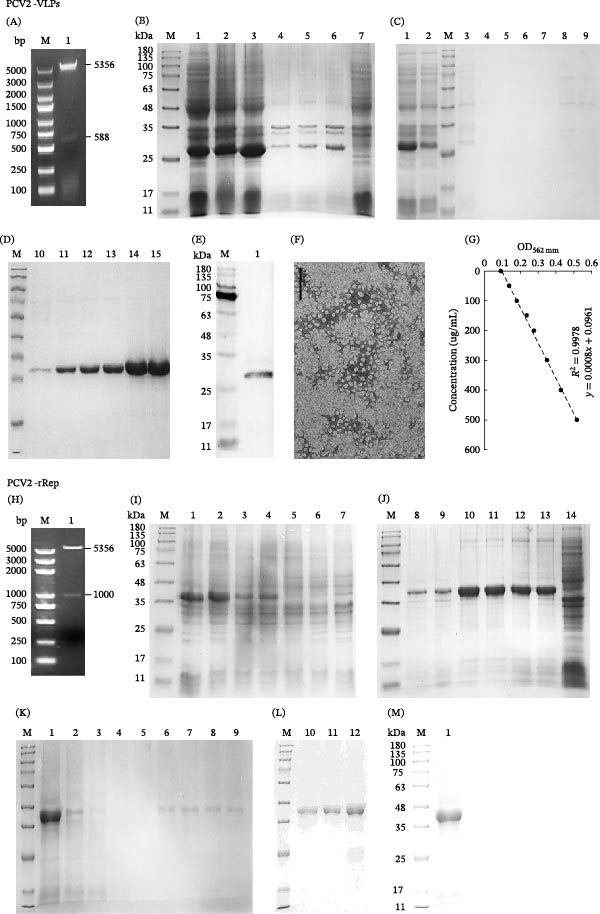
Preparation and characterization of PCV2 rRep protein and virus‐like particles (VLPs). PCV2‐VLPs: (A) Restriction enzyme digestion analysis of the recombinant plasmid. M, DNA molecular weight marker; lane 1, recombinant plasmid pET28a‐Cap2dΔ41. (B) SDS‐PAGE analysis of Cap2dΔ41 protein expression under different induction conditions. M, protein molecular weight marker; lane 1, supernatant of recombinant bacteria induced with 1.0 mM IPTG at 16°C; lane 2, supernatant of recombinant bacteria induced with 1.0 mM IPTG at 25°C; lane 3, supernatant of recombinant bacteria induced with 1.0 mM IPTG at 37°C; lane 4, pellet of recombinant bacteria induced with 1.0 mM IPTG at 16°C; lane 5, pellet of recombinant bacteria induced with 1.0 mM IPTG at 25°C; lane 6, pellet of recombinant bacteria induced with 1.0 mM IPTG at 37°C; lane 7, pET28a(+) empty vector control. (C, D) SDS‐PAGE analysis of Ni‐affinity purification of Cap2dΔ41 protein expressed at 37°C with 1.0 mM IPTG. Lane 1, supernatant of recombinant bacteria; lane 2, pellet of recombinant bacteria; M, protein molecular weight marker; lane 3, flow‐through fraction; lanes 4–6, 50 mM imidazole fractions; lanes 7–9, 100 mM imidazole fractions; lanes 10–12, 250 mM imidazole fractions; and lanes 13–15, 500 mM imidazole fractions. (E) Western blot analysis of purified Cap2dΔ41 protein. M, protein molecular weight marker; lane 1, Cap2dΔ41 protein. (F) Transmission electron microscopy image of assembled PCV2 VLPs. (G) Standard curve generated using the BCA protein assay kit. PCV2‐rRep: (H) Restriction enzyme digestion analysis of the recombinant plasmid. M, DNA molecular weight marker; lane 1, recombinant plasmid pET28a‐rRep. (I, J) SDS‐PAGE analysis of rRep protein expression under different induction conditions. M, protein molecular weight marker; lane 1, supernatant of recombinant bacteria induced with 0.5 mM IPTG at 16°C; lane 2, supernatant of recombinant bacteria induced with 1.0 mM IPTG at 16°C; lane 3, supernatant of recombinant bacteria induced with 0.5 mM IPTG at 25°C; lane 4, supernatant of recombinant bacteria induced with 1.0 mM IPTG at 25°C; lane 5, supernatant of recombinant bacteria induced with 0.5 mM IPTG at 37°C; lane 6, supernatant of recombinant bacteria induced with 1.0 mM IPTG at 37°C; lane 7, supernatant of pET28a(+) empty vector control; lane 8, pellet of recombinant bacteria induced with 0.5 mM IPTG at 16°C; lane 9, pellet of recombinant bacteria induced with 1.0 mM IPTG at 16°C; lane 10, pellet of recombinant bacteria induced with 0.5 mM IPTG at 25°C; lane 11, pellet of recombinant bacteria induced with 1.0 mM IPTG at 25°C; lane 12, pellet of recombinant bacteria induced with 0.5 mM IPTG at 37°C; lane 13, pellet of recombinant bacteria induced with 1.0 mM IPTG at 37°C; and lane 14, pellet of pET28a(+) empty vector control. (K, L) SDS‐PAGE analysis of Ni‐affinity purification of rRep protein expressed at 16°C with 1.0 mM IPTG. M, protein molecular weight marker; lane 1, supernatant of recombinant bacteria; lane 2, pellet of recombinant bacteria; lane 3, flow‐through fraction; lanes 4–5, 50 mM imidazole fractions; lanes 6–7, 100 mM imidazole fractions; lanes 8–9, 250 mM imidazole fractions; and lanes 10–12, 500 mM imidazole fractions. (M) Western blot analysis of purified rRep protein. M, protein molecular weight marker; lane 1, rRep protein.

The recombinant plasmids pET28a‐Cap2dΔ41 and pET28a‐rRep were confirmed by restriction enzyme digestion analysis (Figure 1A,H) and then expressed in *E. coli* BL21 (DE3). SDS‐PAGE and Western blotting showed that rRep (~39 kDa) and Cap2dΔ41 (~27 kDa) were successfully expressed, mainly in the soluble fractions under the selected induction conditions, and were subsequently purified by Ni^2+^‐affinity chromatography with stepwise imidazole elution (Figure 1B–E,I–M). TEM indicated that purified Cap2dΔ41 self‐assembled in vitro into VLPs with uniform morphology and an average diameter of ~22 nm (Figure 1F). A BCA standard curve was generated and used for protein quantification (Figure 1G). These antigens supported the subsequent establishment of the VLPs‐CLIA and rRep‐CLIA.

### 3.2. Establishment of CLIA Methods and Optimization of Key Parameters

**Figure 2:**
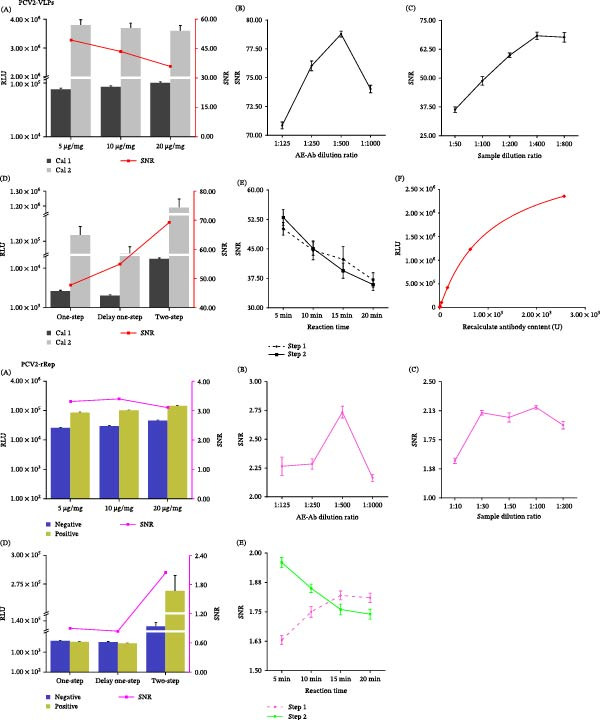
Optimization of key parameters and standard curves for VLPs‐CLIA and rRep‐CLIA. (A) Antigen coupling ratio to magnetic beads; (B) working concentration of AE‐labeled antibody; (C) serum sample dilution factor; and (D) comparison of different reaction formats. (E) Optimization of incubation time for the two‐step reaction format. (F) Quantitative standard curve for VLPs‐CLIA.

Using the prepared VLPs and rRep protein, two fully automated magnetic particle–based CLIAs (VLPs‐CLIA and rRep‐CLIA) were established. Key parameters, including the coated antigen, antigen‐to‐bead coupling ratio, AE‐labeled secondary antibody dilution, serum dilution, reaction format, and incubation time, were systematically optimized (Figure 2A–E). For VLPs‐CLIA, the final optimized conditions were PCV2 Cap VLPs, 5 μg/mg antigen‐to‐bead coupling, 1:400 serum dilution, 1:500 AE‐labeled secondary antibody dilution, and a two‐step reaction with 5 min of incubation in each step. For rRep‐CLIA, the final optimized conditions were recombinant Rep protein, 10 μg/mg antigen‐to‐bead coupling, 1:100 serum dilution, 1:500 AE‐labeled secondary antibody dilution, and a two‐step reaction with 15 min of incubation in step 1 and 5 min in step 2. These conditions are also summarized in Table 2. Standard curves were constructed using serially diluted positive sera, and the VLPs‐CLIA standard curve showed good linearity, with *R* ^2^ > 0.999 (Figure 2F and Table 3).

**Table 2:** Final optimized working conditions for VLPs‐CLIA and rRep‐CLIA.

Parameter	VLPs‐CLIA	rRep‐CLIA
Coated antigen	PCV2 Cap VLPs	Recombinant Rep protein
Antigen‐to‐bead coupling ratio	5 µg/mg	10 µg/mg
Serum dilution	1:400	1:100
AE‐labeled secondary antibody dilution	1:500	1:500
Reaction format	Two‐step	Two‐step
Incubation time per step	5 min (step 1); 5 min (step 2)	15 min (step 1); 5 min (step 2)
Optimization criterion	Signal‐to‐noise ratio	Signal‐to‐noise ratio

**Table 3:** Standard‐curve data for PCV2 Cap antibody detection by VLPs‐CLIA.

Standard/control	Dilution	S/P	Mean RLU	Back‐calculated antibody concentration (U)
S1	1:102,400	0.09	7656	0.00
S2	1:25,600	0.38	30,365	10.10
S3	1:6400	1.21	106,865	39.37
S4	1:1600	2.19	422,712	162.38
S5	1:400	2.59	1,232,774	634.81
S6	1:100	2.68	2,356,189	2567.94

Abbreviation: S/P, sample‐to‐positive ratio.

### 3.3. Analytical Performance

**Figure 3:**
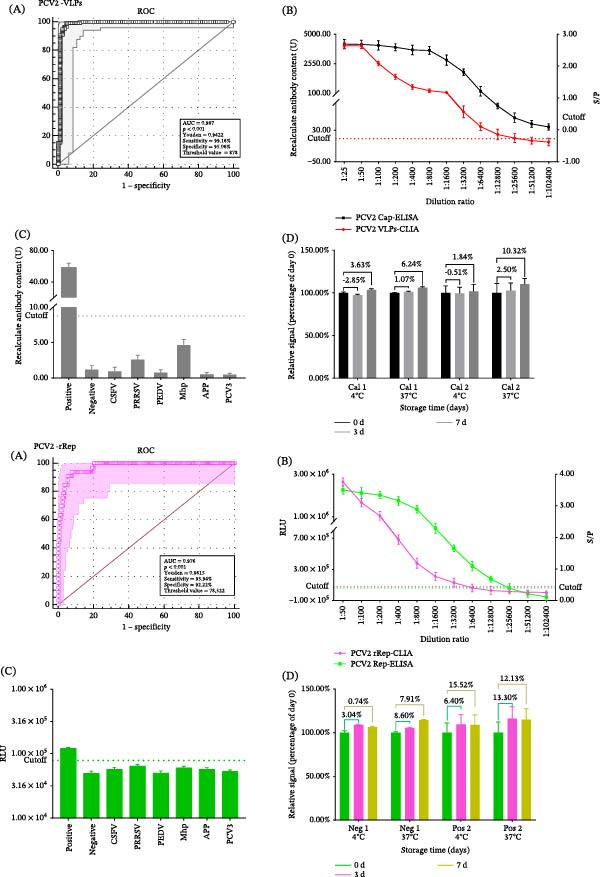
Analytical performance of VLPs‐CLIA and rRep‐CLIA. (A) Receiver operating characteristic (ROC) curve analysis for determination of cut‐off values. (B) Comparison of analytical sensitivity between CLIA methods and ELISA. (C) Specificity evaluation against sera positive for other common swine pathogens. (D) Accelerated stability analysis of VLPs‐CLIA and rRep‐CLIA. Results are presented as relative signal values normalized to Day 0 (100%) to enable direct comparison of signal retention between the two assays.

ROC analysis yielded cut‐offs of 8.78 U for VLPs‐CLIA and 78,522 RLU for rRep‐CLIA, with Youden indices of 0.9422 and 0.8615, respectively (Figure 3A). Samples with values > 8.78 U were considered positive by VLPs‐CLIA, whereas samples with signals > 78,522 RLU were considered positive by rRep‐CLIA. Analytical sensitivity showed that VLPs‐CLIA detected positivity up to a serum dilution of 1:25,600, approximately twofold higher than the reference Cap‐ELISA (1:12,800). For rRep‐CLIA, the detection limit was 1:6400 (Figure 3B). No cross‐reactivity was observed with the tested heterologous pathogen‐positive sera, including CSFV, PRRSV, PEDV, Mhp, APP, and PCV3, under the conditions of the present study (Figure 3C). For a clearer presentation, the negative control shown in Figure 3C was a confirmed PCV2‐negative pig serum sample. Potential PCV1‐related cross‐reactivity could not be evaluated in the present study. The observed intra‐assay and interassay CVs of the two CLIAs ranged from 2.83% to 6.94% (Tables 4–7), indicating good assay precision under the conditions evaluated in this study. These CV values are reported as the observed analytical performance rather than as a predefined universal acceptance criterion. In the accelerated stability test, stability was assessed using relative signal values normalized to Day 0 (set as 100%) to facilitate direct comparison between assays with different absolute signal ranges. Under this ratio‐based presentation, both assays remained stable, with signal variation remaining within 15% after 7 days (Figure 3D). Parallel testing with the reference Cap‐ELISA and Rep‐ELISA methods demonstrated high diagnostic agreement. Agreement with the reference ELISAs was high: VLPs‐CLIA vs. Cap‐ELISA, 95.68% (95% CI: 93.09%–97.32%) with Cohen’s κ = 0.876 (95% CI: 0.816–0.935; *p*  < 0.001) (Table 8); rRep‐CLIA vs. Rep‐ELISA, 94.86% (95% CI: 92.12%–96.69%) with Cohen’s κ = 0.846 (95% CI: 0.779–0.913; *p*  < 0.001) (Table 9).

**Table 4:** Intra‐assay reproducibility results of the VLPs‐CLIA method.

Serum number	Serum sample concentration (U)					Mean (U)	SD	CVs (%)
1	2.54	2.60	2.64	2.52	2.82	2.66	0.12	4.39
	2.75	2.77	2.72	2.48	2.72			
2	19.51	18.44	18.76	19.22	19.34	18.74	0.82	4.40
	19.81	17.85	19.14	18.12	17.20			
3	68.79	79.72	72.37	80.64	81.92	77.45	4.66	6.02
	82.12	77.72	79.27	79.87	72.11			
4	126.75	121.60	110.29	114.86	117.47	120.40	5.16	4.28
	126.39	123.15	118.54	122.76	122.22			

**Table 5:** Interassay reproducibility results of the VLPs‐CLIA method.

Serum number	Different batches of magnetic beads						Mean (U)	SD	CVs (%)
	B1		B2		B3				
1	2.41	2.31	2.42	2.13	2.48	2.52	2.38	0.17	6.94
	2.54	2.23	2.20	2.35	2.64	2.53			
2	18.41	19.73	19.37	18.70	19.30	19.56	19.13	0.76	3.95
	17.75	19.53	18.38	17.42	19.39	19.90			
3	80.17	86.80	77.09	84.30	84.23	75.36	80.26	4.11	5.12
	80.77	75.12	79.23	81.23	82.23	77.01			
4	125.72	122.03	127.54	131.24	125.13	117.13	124.93	4.52	3.62
	119.01	120.64	130.03	124.08	129.02	122.45			

**Table 6:** Intra‐assay reproducibility results of the rRep‐CLIA method.

Serum number	RLU					Mean RLU	SD	CVs (%)
1	25,500	25,262	25,881	24,052	26,128	25,382.30	717.37	2.83
	25,271	26,269	25,906	25,111	24,443			
2	65,551	63,728	65,873	60,676	61,400	63,383.10	2026.21	3.20
	62,742	60,335	65,134	63,657	64,735			
3	100,474	110,219	111,269	101,672	101,402	105,239.50	4108.65	3.90
	104,588	111,086	104,465	103,832	103,388			
4	134,643	125,643	129,425	128,255	124,555	129,130.80	3364.06	2.61
	125,306	133,125	131,691	129,715	128,950			

**Table 7:** Interassay reproducibility results of the rRep‐CLIA method.

Serum number	Different batches of magnetic beads						Mean RLU	SD	CVs (%)
	B1		B2		B3				
1	20,554	22,220	23,443	26,371	22,766	24,493	24,174.58	1582.54	6.55
	25,255	25,753	24,718	22,948	26,889	24,685			
2	67,254	63,741	63,532	68,236	66,316	64,735	65,570.67	2517.72	3.84
	66,017	64,727	69,849	61,551	67,239	63,651			
3	110,684	104,588	111,050	101,386	117,312	100,630	106,867.75	6301.01	5.90
	108,423	100,796	110,219	114,251	101,672	101,402			
4	128,569	131,111	129,125	125,271	130,427	131,442	130,172.17	3720.51	2.86
	131,795	125,634	134,574	125,566	135,284	133,268			

**Table 8:** Agreement results between the VLPs‐CLIA and Cap‐ELISA methods.

Method type		PCV2 VLPs‐CLIA		
		Positive	Negative	Total
PCV2 Cap‐ELISA	Positive	279	10	289
	Negative	6	75	81
	Total	285	85	370

*Note:* Overall agreement = 95.68% (354/370; 95% CI, 93.09%–97.32%); Cohen’s κ = 0.876 (95% CI, 0.816–0.935).

**Table 9:** Agreement results between the rRep‐CLIA and Rep‐ELISA methods.

Method type		PCV2 rRep‐CLIA		
		Positive	Negative	Total
PCV2 Rep‐ELISA	Positive	69	10	79
	Negative	9	282	291
	Total	78	292	370

*Note*: Overall agreement = 94.86% (351/370; 95% CI: 92.12%–96.69%); Cohen’s κ = 0.846 (95% CI: 0.779–0.913).

### 3.4. Evaluation of Clinical Application

In longitudinal monitoring, Cap antibody levels in Cap‐vaccinated farms (A and C) increased over time as measured by VLPs‐CLIA and Cap‐ELISA (Figure 4, PCV2‐Cap A and C). During the same period, Rep antibody signals remained low in these farms by both rRep‐CLIA and Rep‐ELISA (Figure 4, PCV2‐Rep A and C).

Figure 4Longitudinal dynamic monitoring of PCV2 antibody responses. Panels show temporal changes in antibody readouts in vaccinated farms (A and C), nonvaccinated farms (F and G), and clinically monitored herds with unknown vaccination backgrounds (B, D, and E), as measured by CLIA and the corresponding reference ELISA. Each point represents the mean antibody readout of all serum samples collected at the indicated sampling time in the corresponding herd/group, and lines connect the time‐point means to illustrate temporal trends. VLPs‐CLIA results are expressed as recalculated antibody content (U), rRep‐CLIA results are expressed as relative light units (RLUs), and the corresponding ELISA results are expressed as S/P values.

**Figure:**
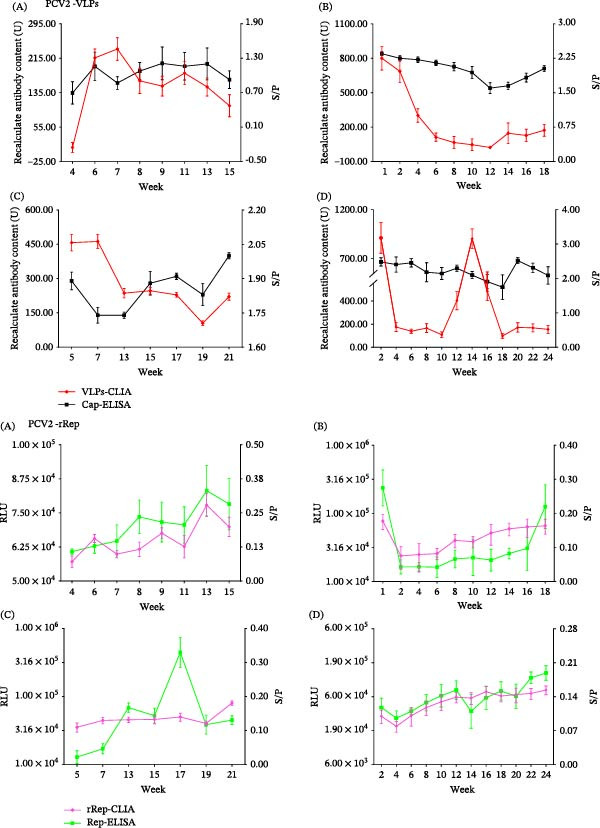

**Figure:**
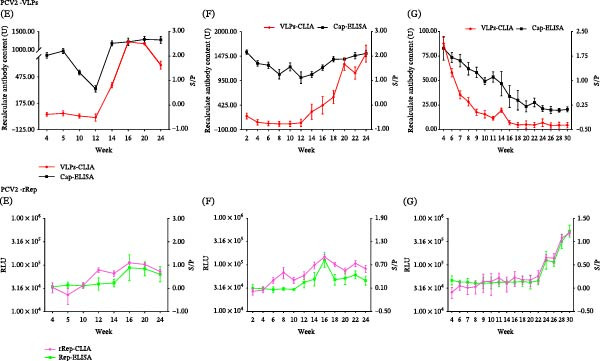

In nonvaccinated farms (F and G), Rep antibody signals were persistently detected by both rRep‐CLIA and Rep‐ELISA (Figure 4, PCV2‐Rep F and G). In clinically monitored herds with unknown vaccination backgrounds (farms B, D, and E), rRep‐CLIA detected Rep antibody signals at varying levels; accordingly, these Rep antibody profiles were interpreted as serological patterns potentially reflecting heterogeneous replication–associated exposure rather than confirmed infection status.

**Figure 5:**
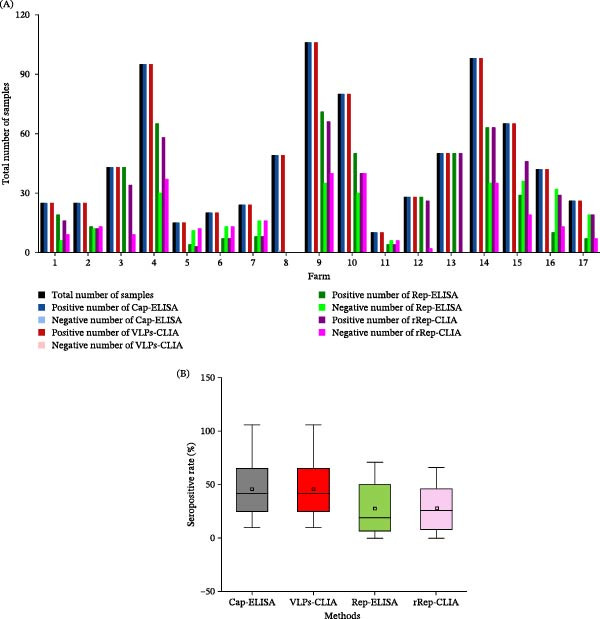
Serological screening results of PCV2 in large‐scale pig farms. (A) Total number of serum samples tested in each farm/group and the corresponding numbers of positive and negative samples obtained by Cap‐ELISA, VLPs‐CLIA, Rep‐ELISA, and rRep‐CLIA. (B) Comparison of seropositive rates determined by the four methods across farms/groups. For each farm/group, the seropositive rate was calculated as the number of positive samples divided by the total number of samples tested in that farm/group (*n*). The sample size (*n*) for each farm/group is the same as that shown in panel A and is also summarized in Table 1.

In large‐scale screening across 14 breeding farms and three slaughterhouses in Hunan Province, Cap antibody seropositivity remained high across breeding farms by both VLPs‐CLIA and Cap‐ELISA (Figure 5B). Results from the rRep‐CLIA were consistent with Rep‐ELISA (Figure 5A), whereas Rep antibody seropositivity varied markedly among farms (Figure 5B). Notably, slaughterhouse sera (farms 15–17) showed the highest Rep antibody seropositivity. Together, these patterns support the use of Cap antibodies for evaluating vaccine‐induced Cap response intensity and Rep antibodies for reflecting replication‐associated pressure at the herd level.

## 4. Discussion

PCV2 has immunosuppressive properties and may facilitate mixed infections in pig populations, posing an ongoing threat to the swine industry [28–30]. In this study, we established two CLIAs based on PCV2 Cap VLPs and Rep and evaluated their utility for serological surveillance using longitudinal and cross‐sectional field samples. Our data showed that VLPs‐CLIA quantitatively reflected vaccine‐induced Cap antibody levels, whereas rRep‐CLIA provided a complementary signal consistent with replication‐associated exposure at the herd level, particularly in Cap‐vaccinated settings. Together, these two assays enabled concurrent assessment of Cap immunity and replication‐associated serological pressure in highly vaccinated populations.

Chemiluminescence‐based assays have already been explored in porcine circovirus diagnostics, particularly for PCV2. Early PCV2‐related CLIA studies focused mainly on direct viral or antigen detection, and later developments largely remained within single‐target formats, including magnetic chemiluminescence platforms for PCV2 antigen testing [31–34]. More recently, antibody‐detection formats have also been reported or publicly disclosed, but these assays have still relied predominantly on single‐antigen strategies based on recombinant PCV2 proteins, with the clearest examples centered on Cap‐related antigens, including competitive, indirect, and automated tube‐based CLIAs [35–37]. Thus, although CLIA has been introduced into PCV2 detection, the current technical landscape remains relatively limited and is still dominated by single‐target, largely Cap‐based designs. Compared with previously reported PCV2 chemiluminescence assays, which have mainly focused on antigen detection or single‐marker Cap serology, the present study establishes an automated magnetic particle–based dual‐marker serological framework by integrating PCV2 Cap VLPs, which preserve conformational epitopes, with rRep for complementary herd‐level interpretation.

Rep is one of the key nonstructural proteins of porcine circoviruses and has been studied mainly in relation to viral replication. In PCV1 and PCV2, Rep/Rep′ has been associated with rolling‐circle replication, host interaction, and, more recently, innate immune antagonism [38–41]. In PCV2, Rep/Rep′ is immunogenic but nonprotective, and its serodiagnostic potential has been explored, including a Rep′‐based iELISA for distinguishing natural infection or whole‐virus immunization from subunit vaccine immunization [42–44]. By contrast, Rep‐related studies in PCV3 remain limited, and serological assay development is still largely dominated by Cap/VLP‐based formats [45, 46]. Thus, the value of the present study lies not in introducing Rep as a new antigenic target but in extending previous PCV2 serological approaches by integrating PCV2 Cap VLPs, a fully automated magnetic particle–based CLIA platform, and dual‐marker (Cap/Rep) analysis within the same workflow and by further evaluating this combined strategy in both longitudinal and cross‐sectional field settings. This design supports more practical herd‐level interpretation of vaccine‐associated Cap responses and replication‐associated serological exposure.

Antigen selection remains a key determinant of both analytical performance and biological interpretability in PCV2 immunoassay development. PCV2 Cap can self‐assemble into VLPs that preserve conformational epitopes resembling native virions, which may improve antibody detection sensitivity [11, 47]. In the present study, high‐purity PCV2 VLPs were produced in *E. coli* and used to establish VLPs‐CLIA. This assay showed approximately twofold higher analytical sensitivity than Cap‐ELISA and high agreement with Cap‐ELISA, supporting its use for quantitative monitoring of vaccine‐induced Cap antibody responses. In parallel, rRep‐CLIA showed good stability and agreement with Rep‐ELISA in the analytical evaluation and field sample testing. Longitudinal monitoring further suggested that Rep antibodies were mainly detected in nonvaccinated herds or herds with suspected infection pressure, supporting rRep serology as a complementary indicator of replication‐associated antigen exposure at the herd level. Taken together, these findings indicate that Cap VLPs and rRep provide distinct but complementary serological information for PCV2 monitoring.

Building on these complementary assay characteristics, this study further examined the value of their combined application. Large‐scale screening showed consistently high Cap seropositivity across farms, suggesting widespread vaccine‐induced immunity, whereas Rep seropositivity varied markedly, with higher levels in slaughterhouse samples. These findings suggest that, even under high vaccine coverage, replication‐associated serological profiles may still differ substantially among herds. Therefore, joint interpretation of Cap and Rep responses may provide a more refined herd‐level assessment by integrating vaccine‐induced Cap response intensity with replication‐associated serological pressure.

Several limitations should also be acknowledged. First, reliance on a dedicated fully automated analyzer may restrict application in resource‐limited settings. Second, longer‐term reagent stability and assay performance across diverse PCV2 variants still require further evaluation. Third, because Rep is relatively conserved between PCV1 and PCV2 [15, 17] and PCV1‐positive sera or antigen was unavailable in the present study, potential PCV1 cross‐reactivity could not be assessed. For this reason, rRep‐CLIA should be interpreted as a complementary indicator of replication‐associated exposure rather than a strictly PCV2‐specific marker, and its interpretation should be combined with Cap profiles, vaccination history, and epidemiological context.

In summary, VLPs‐CLIA and rRep‐CLIA support sensitive monitoring of vaccine‐induced Cap responses and assessment of replication‐associated serological pressure, respectively. By integrating structural‐antigen immunity and replication‐associated exposure within the same analytical framework, this dual‐indicator strategy may improve PCV2 serological surveillance and support more informed prevention and control decisions.

## 5. Conclusions

In this study, fully automated CLIAs based on PCV2 VLPs and the Rep protein were established. The VLPs‐CLIA enables sensitive evaluation of vaccine‐induced Cap antibody responses, whereas the rRep‐CLIA provides complementary serological evidence of replication‐associated exposure at the herd level, particularly in Cap‐vaccinated populations. The combined application of these two assays provides a practical dual‐marker tool for integrated assessment of PCV2 serological immune status (Cap response intensity) and replication‐associated serological pressure (Rep) in pig herds.

## Author Contributions

**Mingxi Gou**: investigation, methodology, formal analysis, writing – original draft. **Xiaoqing Song**: investigation, methodology, formal analysis, writing – original draft. **Jie Fan**: visualization, data curation. **Zhixiong Chen**: investigation, methodology. **Zihao Kuang**: methodology, data curation. **Mengyao Lu**: data curation. **Zhongxin Fan**: funding acquisition, project administration, writing – review and editing. **Meng Ge**: conceptualization, funding acquisition, project administration, supervision, writing – review and editing.

## Funding

This study was supported by the National Natural Science Foundation of China (Grant 32072871) and the Hunan Provincial Key Research and Development Program (Grant 2023NK2017).

## Ethics Statement

The animal study was reviewed and approved by the Animal Experiments Ethics Committee of Hunan Agricultural University (Approval Number 2024‐132). The Experimental Animal Care and Use Guidelines from the Ministry of Science and Technology of China (MOST‐2011‐02) were strictly followed.

## Conflicts of Interest

The authors declare no conflicts of interest.

## Data Availability

The data that support the findings of this study are available upon request from the corresponding author. The data are not publicly available due to privacy or ethical restrictions.
